# Pattern, Outcomes, and Factors Associated With Mortality Among Children Admitted to a Pediatric Intensive Care Unit at King Salman Armed Forces Hospital, Tabuk, Saudi Arabia: A Retrospective Cohort Study

**DOI:** 10.7759/cureus.110804

**Published:** 2026-06-13

**Authors:** Amjad M Altunusi, Laila AlBishi, Abdulaziz Malki, Mustafa M Altoonisi, Wessam Soliman, Mohammed Kamal, Ahmed M Karkar

**Affiliations:** 1 Pediatric Department, King Salman Armed Forces Hospital, Tabuk, SAU; 2 Pediatric Endocrinology, Faculty of Medicine, University of Tabuk, Tabuk, SAU; 3 Pediatric Intensive Care Unit, King Salman Armed Forces Hospital, Tabuk, SAU

**Keywords:** admission source, case mix, mechanical ventilation, pediatric intensive care, picu mortality, retrospective cohort, saudi arabia

## Abstract

Background: Pediatric intensive care units (PICUs) provide advanced care for critically ill children with heterogeneous medical and surgical conditions. Contemporary local data on PICU case mix, outcomes, and factors associated with mortality remain limited in northwestern Saudi Arabia and are essential for benchmarking performance and guiding quality-improvement initiatives.

Objective: To describe admission patterns, clinical outcomes, and factors associated with in-PICU mortality among children admitted to the PICU at King Salman Armed Forces Hospital (KSAFH), Tabuk, Saudi Arabia.

Methods: We conducted a retrospective cohort study of all children aged 1 day to 14 years admitted to the PICU at KSAFH between 1 January and 31 December 2024. Data on demographics, admission source, diagnoses, interventions, and outcomes were extracted from electronic medical records using a structured data-collection form. Continuous variables were summarized as median and interquartile range (IQR); categorical variables were summarized as frequencies and percentages. Bivariate comparisons used the chi-square or Fisher exact test for categorical variables and the Mann-Whitney U test for continuous variables. Odds ratios (ORs) with 95% confidence intervals (CIs) were calculated for clinically relevant exposures. Because only eight deaths occurred, multivariable logistic regression was not performed in order to avoid model overfitting. A two-sided p < 0.05 was considered statistically significant.

Results: Of 318 PICU admissions during the study period, 310 had complete outcome data and were analyzed. The cohort was nearly evenly distributed by sex (51.3% female, 48.7% male), with a median age of 3 years and a median weight of 13 kg (IQR 6-23). The most common admission source was the emergency department (62.6%), followed by inpatient wards (28.4%); transport and perioperative pathways together accounted for 9.0% of admissions. Respiratory and metabolic/other conditions were the leading admission categories (35.5% and 37.4%, respectively); the most frequent admission diagnoses were asthma exacerbation (16.5%), diabetic ketoacidosis (13.2%), acute bronchiolitis (9.7%), and pneumonia (4.5%). Thirty-two children (10.3%) required invasive mechanical ventilation (median duration 6.5 days; IQR 3-10). The overall in-PICU mortality rate was 2.6% (8/310), and the median PICU length of stay was three days (IQR 1-6). Mechanical ventilation was strongly associated with mortality (18.8% vs. 0.7%; OR 31.85, 95% CI 6.12-165.83; p < 0.001), as was admission via transport or perioperative pathways relative to admission from the emergency department or wards (10.7% vs. 1.8%; OR 6.65, 95% CI 1.50-29.46; p = 0.027). Age group, sex, and disease category were not significantly associated with mortality, although infants accounted for half of all deaths.

Conclusions: In this contemporary cohort from northwestern Saudi Arabia, overall PICU mortality was low (2.6%). Deaths were concentrated among children requiring invasive mechanical ventilation and those admitted through transport or perioperative pathways. These findings support strengthening early risk recognition, optimizing pre-PICU stabilization and inter-facility transfer pathways, and integrating standardized severity-of-illness scoring into routine PICU practice.

## Introduction

Pediatric intensive care units (PICUs) provide advanced care for critically ill children requiring continuous monitoring and life-support interventions. Although only a minority of hospitalized children require PICU admission, this population contributes disproportionately to morbidity, mortality, and healthcare utilization [1,2]. Contemporary local data remain important because case mix, referral pathways, and thresholds for PICU admission vary across centers [1,3,4].

This is particularly relevant in units that function not only as sites of invasive organ support but also as high-dependency environments for close observation, noninvasive respiratory support, metabolic stabilization, and perioperative monitoring. Recognizing this broader operational role is essential for interpreting crude mortality rates, resource use, and admission patterns.

Respiratory illness, infection, trauma, metabolic derangement, and postoperative care are frequently represented in PICU cohorts [1,2,4]. Several studies have linked mortality to illness severity, need for mechanical ventilation, younger age, and admission pathway [2,5-10]. The present study aimed to evaluate admission patterns, clinical outcomes, and factors associated with mortality in a tertiary-care PICU in northwest Saudi Arabia.

## Materials and methods

Study design and setting

This retrospective cohort study was conducted in the Pediatric Intensive Care Unit of King Salman Armed Forces Hospital, Tabuk, Saudi Arabia, over a one-year period from 1 January 2024 to 31 December 2024.

Study population

All children aged 1 day to 14 years admitted to the PICU during the study period were screened for inclusion.

Inclusion and exclusion criteria

Inclusion criteria were all PICU admissions during the study period with complete outcome data. Exclusion criteria were incomplete outcome data, unknown disposition, and short-observation admissions not meeting the unit admission threshold for full PICU analytic inclusion.

Data source and collection

Data were extracted retrospectively from the hospital electronic medical record and the PICU admission registry using a structured data-collection sheet prepared for the study. Collected variables included age, sex, weight, admission source, disease category, admission diagnosis, reason for admission, use of invasive mechanical ventilation, duration of ventilation, length of stay, code status, disposition, and survival at PICU discharge. Where documented in the registry, noninvasive and advanced respiratory support modalities such as high-flow nasal cannula, continuous positive airway pressure, bilevel positive airway pressure/noninvasive ventilation, and high-frequency oscillatory ventilation were reviewed descriptively to better characterize actual PICU interventions. Disability at discharge was based on routine clinical chart documentation rather than a validated functional outcome instrument.

PICU admission practice

At the study center, PICU admission is based on clinical judgment and local unit practice rather than a single standardized protocol score. Children are typically admitted for invasive or noninvasive respiratory support, hemodynamic instability or close cardiovascular monitoring, metabolic stabilization such as diabetic ketoacidosis management, postoperative observation, neurologic monitoring, or close observation when deterioration risk is judged to be high. Accordingly, the cohort includes both high-acuity children requiring organ support and lower-acuity children needing intensive monitoring.

Statistical analysis

Continuous variables were summarized as median and interquartile range (IQR), and categorical variables as frequencies and percentages. Bivariate comparisons were performed using the chi-square test or Fisher exact test for categorical variables and the Mann-Whitney U test for continuous variables. Odds ratios with 95% confidence intervals were calculated for key mortality-associated factors. A two-sided p-value of less than 0.05 was considered statistically significant. Because only eight deaths occurred, multivariable regression and tree-based modeling were not pursued, as such approaches would have risked overfitting and unstable effect estimates.

Outcomes

The primary outcome was in-PICU mortality, defined as death from any cause occurring during the index PICU admission. Secondary outcomes included PICU length of stay, the requirement for invasive mechanical ventilation and its duration, and survival with disability (mild, moderate, or severe) versus survival without disability, as documented in the discharge summary.

Ethical considerations

The study was conducted in accordance with the Declaration of Helsinki and was approved by the institutional review board of King Salman Armed Forces Hospital, Tabuk, Saudi Arabia. Because the study involved a retrospective review of de-identified medical records, the requirement for written informed consent was waived. All data were stored on password-protected institutional devices and accessed only by authorized members of the research team.

## Results

A total of 318 PICU admissions were recorded during the study period. Eight admissions were excluded because of incomplete outcome data, leaving 310 admissions in the final analysis. The cohort had a near-equal sex distribution, and most patients were younger than five years. Admissions originated predominantly from the emergency department, followed by inpatient wards and external transport or perioperative pathways. Thirty-two patients required invasive mechanical ventilation.

The descriptive profile also showed that PICU utilization extended beyond invasive ventilation. Immediate reasons for admission included respiratory support, close observation, diabetic ketoacidosis management, shock, postoperative care, phototherapy, line insertion, fluid overload management, exchange transfusion, infusion-based care, electrolyte imbalance, trauma code, and code blue presentations. These findings indicate that the unit functioned as a mixed-acuity PICU/high-dependency environment during the study period.

Respiratory disease was the leading diagnostic category, followed by other or metabolic conditions, surgical admissions, nephrology, shock or multiple organ dysfunction syndrome, neurology, and cardiac disease. The most common specific diagnoses were asthma exacerbation, diabetic ketoacidosis, acute bronchiolitis, chronic kidney disease, and postoperative care.

Among recorded respiratory support interventions in the registry, the cohort included not only invasive mechanical ventilation but also documented use of high-flow nasal cannula, continuous positive airway pressure, bilevel positive airway pressure/noninvasive ventilation, and high-frequency oscillatory ventilation in selected patients. These intervention patterns help explain why many children were admitted despite not requiring conventional invasive ventilation.

Overall mortality was 2.6%. Most patients survived without documented disability, and one patient survived with mild disability. On bivariate analysis, invasive mechanical ventilation and admission source were significantly associated with mortality. Age group, sex, disease category, unplanned admission, length of stay, and weight were not statistically significant, although infants accounted for a disproportionate share of deaths.

Table 1 shows the baseline demographic, clinical, and operational characteristics of children admitted to the hospital. Table 2 shows the bivariate analysis of factors associated with in-PICU mortality. Table 3 shows clinical details of non-survivors. 

**Table 1:** Baseline demographic, clinical, and operational characteristics of children admitted to the PICU at King Salman Armed Forces Hospital, Tabuk, in 2024 (n = 310). IQR, interquartile range; MODS, multiple organ dysfunction syndrome; OR, operating room; MEDIVAC, medical evacuation; PICU, pediatric intensive care unit. *Among the 32 mechanically ventilated children.

Characteristic	Value
Sex, n (%)	
Female	159 (51.3)
Male	151 (48.7)
Age group, n (%)	
<30 days (neonates)	33 (10.6)
1–12 months	62 (20.0)
1–5 years	88 (28.4)
6–10 years	69 (22.3)
11–14 years	58 (18.7)
Body weight, kg, median (IQR)	13 (6–23)
Weight category, n (%)	
<3 kg	34 (11.0)
3–10 kg	84 (27.1)
10–25 kg	135 (43.5)
25–45 kg	41 (13.2)
Adult-size / obese	16 (5.2)
Source of admission, n (%)	
Emergency department	194 (62.6)
Inpatient ward	88 (28.4)
OR (elective booking)	15 (4.8)
Local transport	8 (2.6)
OR (emergency)	4 (1.3)
MEDIVAC	1 (0.3)
Unplanned admission, n (%)	27 (8.7)
Disease category, n (%)	
Respiratory	110 (35.5)
Other / metabolic	116 (37.4)
Surgical	32 (10.3)
Nephrology	21 (6.8)
Shock / MODS	12 (3.9)
Neurologic	11 (3.5)
Cardiac	8 (2.6)
Invasive mechanical ventilation, n (%)	32 (10.3)
Duration of ventilation, days, median (IQR)*	6.5 (3–10)
Code status, n (%)	
Full code	298 (96.1)
Do-not-resuscitate (DNR)	12 (3.9)
PICU length of stay, days, median (IQR)	3 (1–6)
Outcome, n (%)	
Survival without disability	301 (97.1)
Survival with mild disability	1 (0.3)
In-PICU death	8 (2.6)
Disposition at PICU discharge, n (%)	
Inpatient ward	286 (92.3)
Home	8 (2.6)
MEDIVAC transfer	8 (2.6)
Mortuary	8 (2.6)

**Table 2:** Bivariate analysis of factors associated with in-PICU mortality (n = 310). CI: confidence interval; ED: emergency department; IQR: interquartile range; OR: odds ratio; PICU: pediatric intensive care unit. p-values for sex, age group, disease category, and admission source were calculated using the Fisher exact test (test statistic not applicable, shown as “—”). For mechanical ventilation, both the chi-square test (χ² = 37.11) and Fisher exact test gave concordant results, and the Fisher p value is reported. The Mann–Whitney U test was used for PICU length of stay (U = 759.5). The OR for the “other (nephro/neuro/shock)” disease grouping was not estimable because no deaths occurred in those subgroups

Variable	Survivors (n = 302)	Non-survivors (n = 8)	OR (95% CI)	Test statistic	p-value
Sex, n (%)			—	—	0.72
Female	154 (51.0)	5 (62.5)			
Male	148 (49.0)	3 (37.5)			
Age group, n (%)			—	—	0.18
<30 days	32 (10.6)	1 (12.5)			
1–12 months	58 (19.2)	4 (50.0)			
1–5 years	88 (29.1)	0 (0.0)			
6–10 years	67 (22.2)	2 (25.0)			
11–14 years	57 (18.9)	1 (12.5)			
Disease category, n (%)			—	—	0.29
Respiratory	108 (35.8)	2 (25.0)			
Other / metabolic	112 (37.1)	4 (50.0)			
Surgical	31 (10.3)	1 (12.5)			
Cardiac	7 (2.3)	1 (12.5)			
Other (nephro/neuro/shock)	44 (14.6)	0 (0.0)			
Mechanical ventilation, n (%)	26 (8.6)	6 (75.0)	31.85 (6.12–165.83)	χ² = 37.11	<0.001
Admission source, n (%)			6.65 (1.50–29.46)	—	0.027
Transport / perioperative	25 (8.3)	3 (37.5)			
ED or inpatient ward	277 (91.7)	5 (62.5)			
PICU length of stay, days, median (IQR)	3 (1–6)	8.5 (2.0–10.5)	—	U = 759.5	0.07

**Table 3:** Clinical profile of the eight non-survivors. d: days; DNR: do-not-resuscitate; ED: emergency department; F: female; LOS: length of stay; M: male; mo: months; MV: mechanical ventilation; OR: operating room; yr: years.

Age	Sex	Admission source	Disease category	Primary diagnosis	MV (days)	PICU LOS (days)	Code status
5 mo	F	ED	Other	Meningitis	No	1	DNR
2 mo	M	Local transport	Cardiac	Congenital heart disease	Yes (2)	2	Full code
9 yr	F	Local transport	Other	Burn	Yes (9)	9	Full code
1 mo	F	Inpatient ward	Respiratory	Apnea	Yes (1)	36	Full code
6 d	F	Emergency OR	Surgical	Post-operative	Yes (1)	2	Full code
10 yr	M	ED	Other	Near-drowning	Yes (13)	12	DNR
4 mo	F	ED	Respiratory	Pneumonia	No	8	DNR
13 yr	M	ED	Other	Leuko- dystrophy	Yes (6)	10	DNR

**Figure 1:**
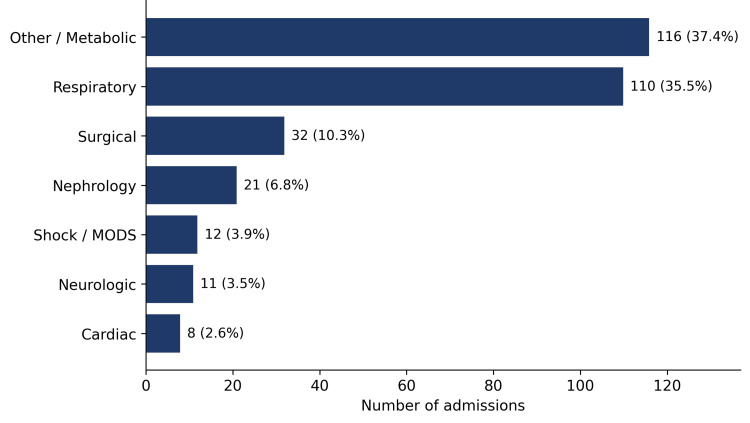
PICU admissions by disease category (n = 310). The most frequent admission categories were “other/metabolic” (37.4%) and respiratory disease (35.5%). MODS, multiple organ dysfunction syndrome.

**Figure 2:**
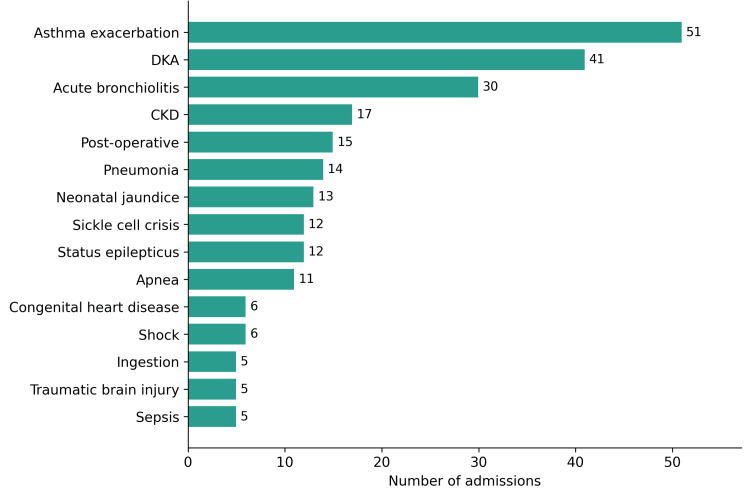
Top 15 admission diagnoses. Asthma exacerbation, diabetic ketoacidosis (DKA), acute bronchiolitis, and chronic kidney disease (CKD) were the most frequent admission diagnoses. Frequencies are absolute counts.

**Figure 3:**
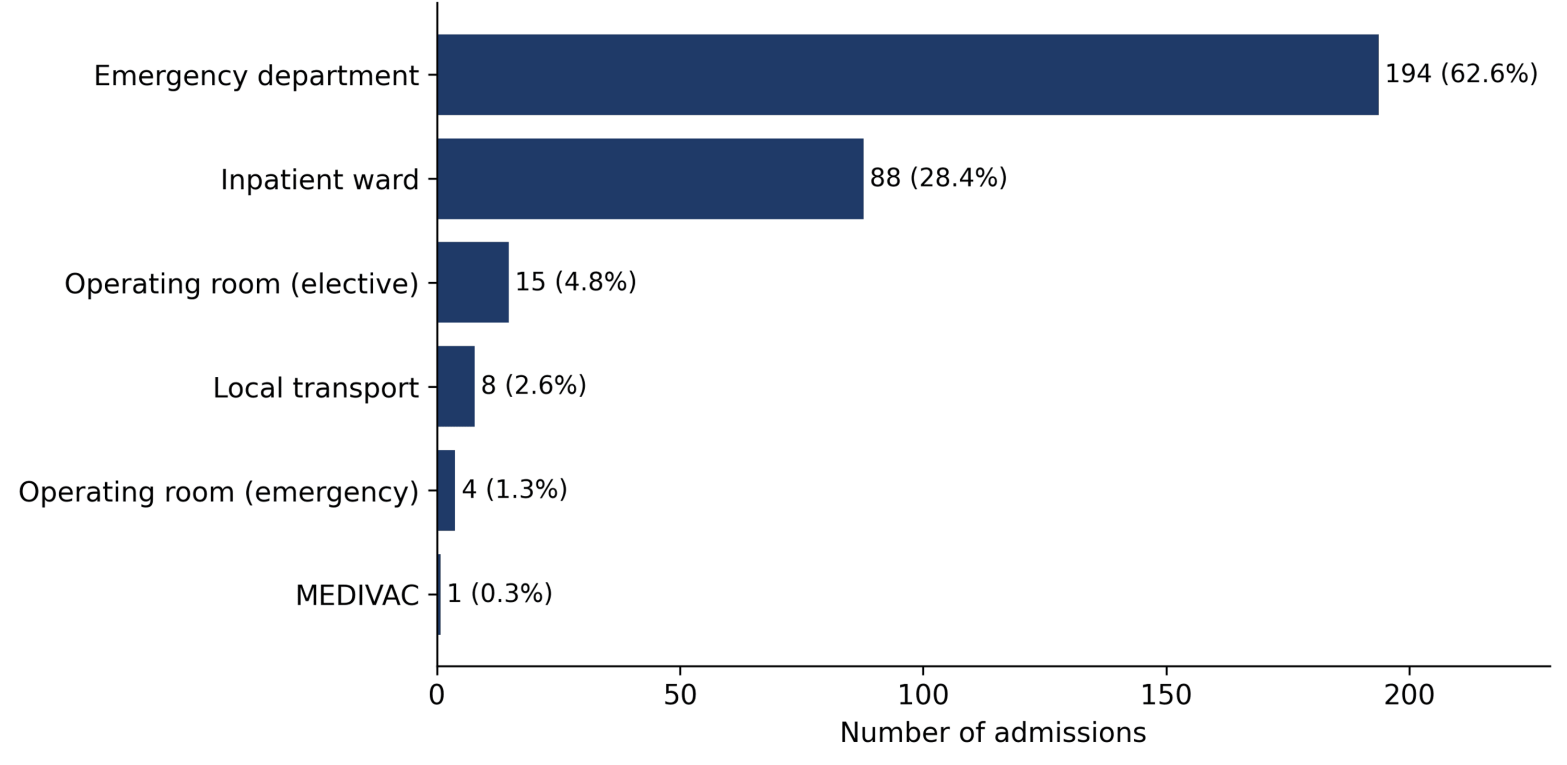
Distribution of PICU admission sources. The emergency department was the dominant source of admission, followed by inpatient wards. OR: operating room

**Figure 4:**
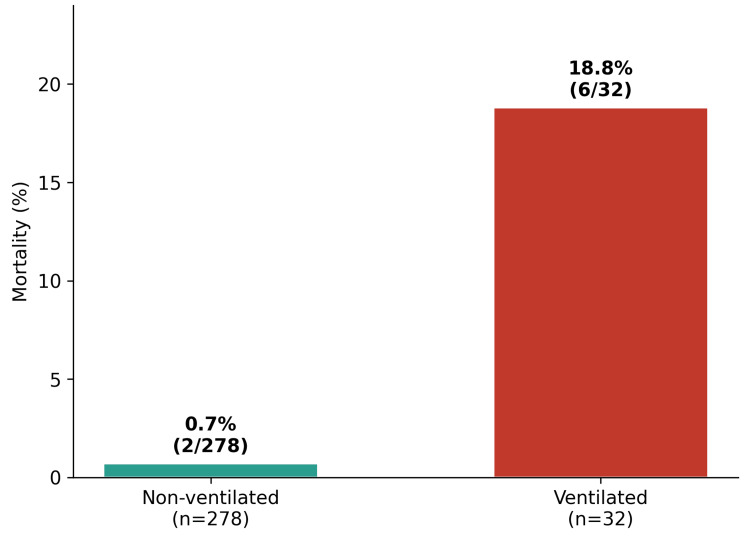
In-PICU mortality stratified by invasive mechanical ventilation status. Ventilated children had a substantially higher case-fatality rate than non-ventilated children. OR: odds ratio; CI: confidence interval

**Figure 5:**
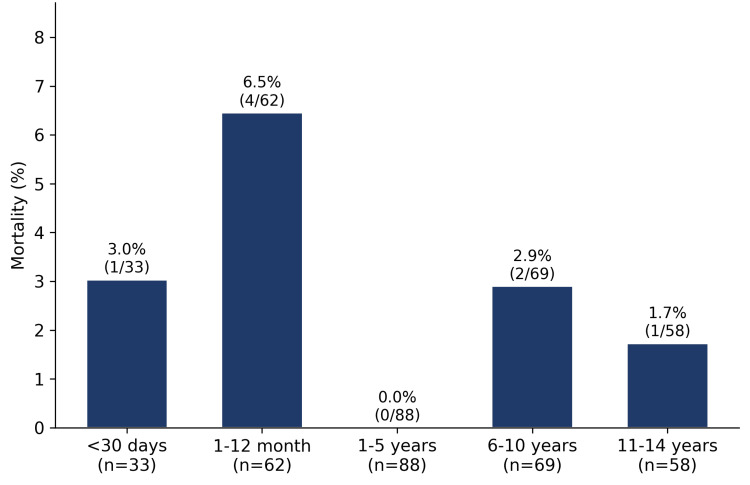
In-PICU mortality by age group. Infants aged 1–12 months had the highest absolute number of deaths, although age group was not statistically associated with mortality.

**Figure 6:**
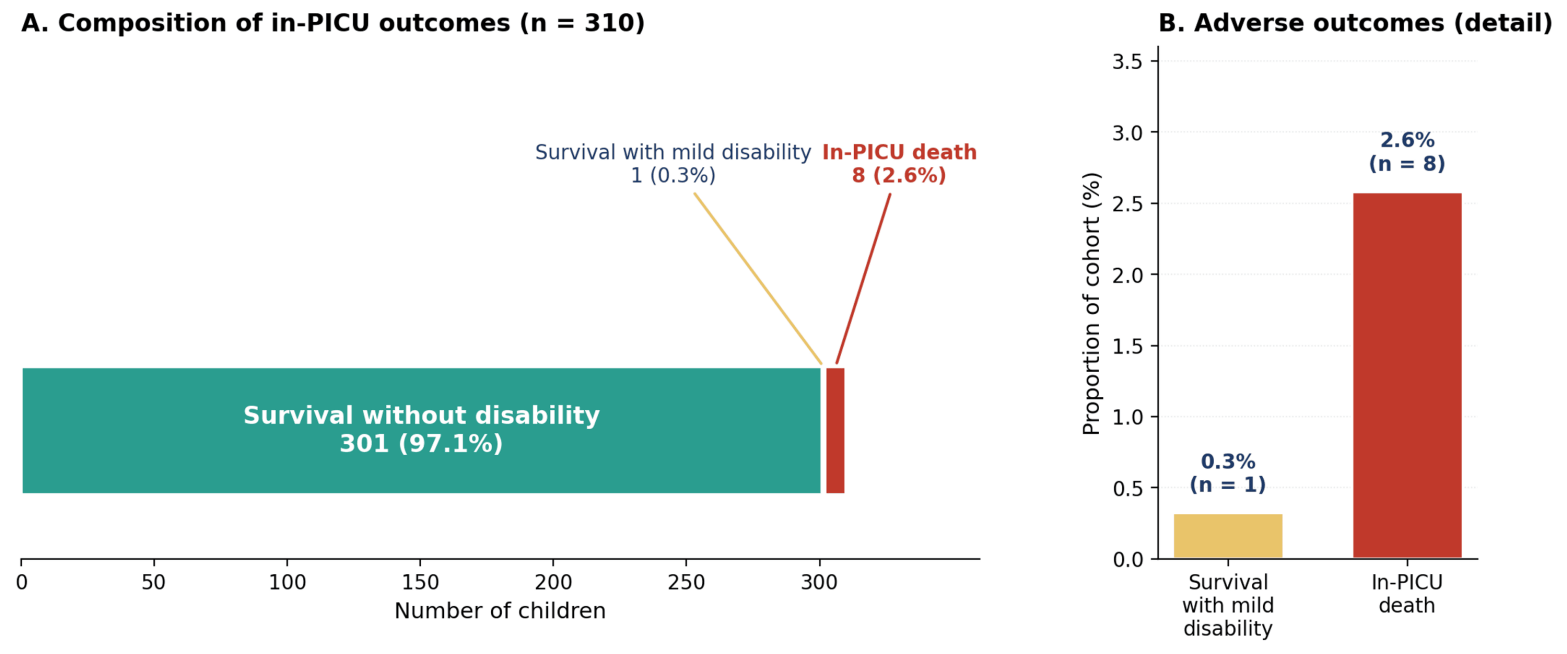
Distribution of in-PICU outcomes (n = 310). The vast majority of children survived without documented disability.

**Figure 7:**
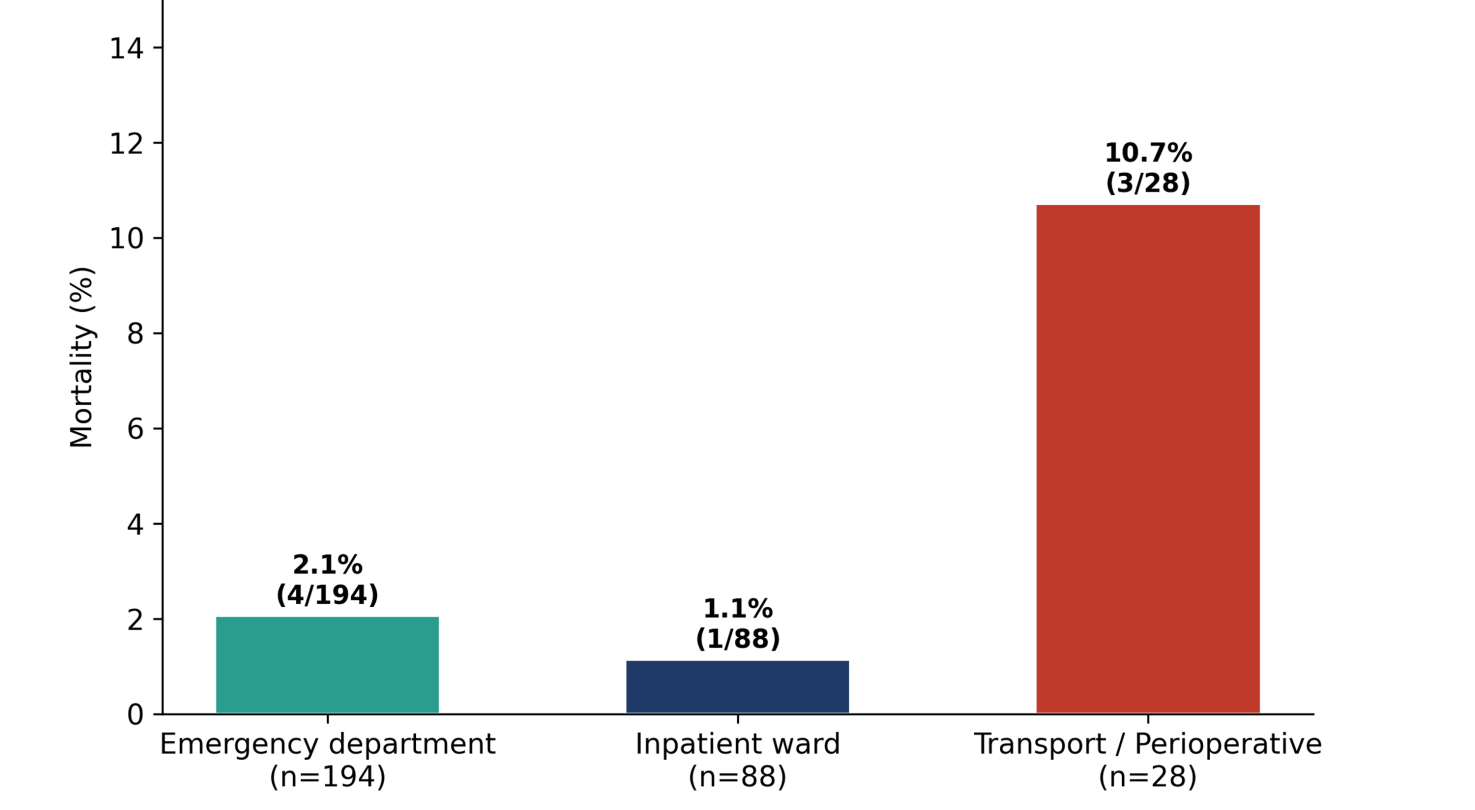
In-PICU mortality stratified by admission source. Children admitted through transport or perioperative pathways had a higher case-fatality rate than those admitted from the emergency department or inpatient wards.

**Figure 8:**
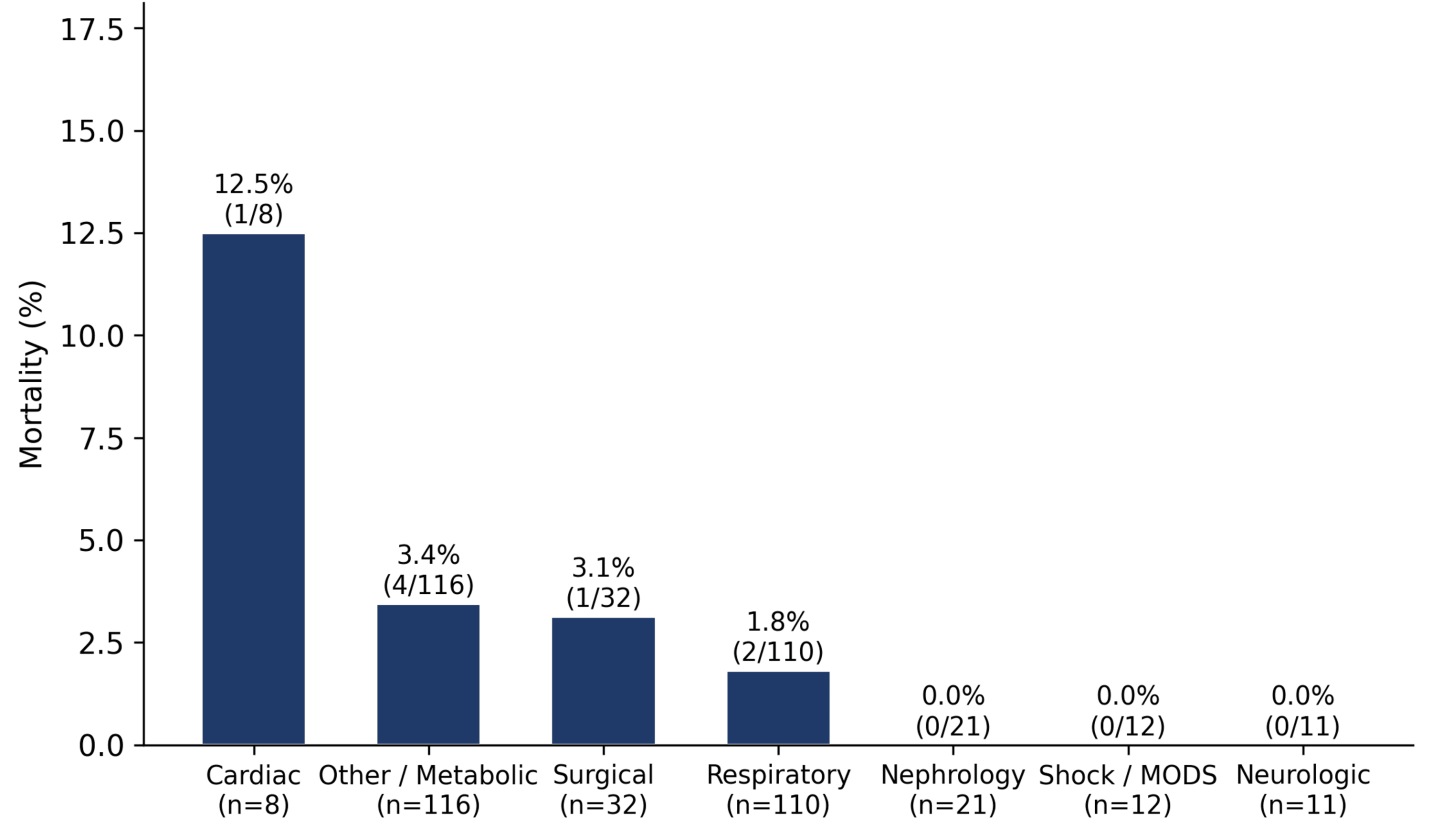
Case-fatality rate by disease category. The cardiac subgroup had the highest case-fatality rate, although the absolute number of cardiac admissions was small.

**Figure 9:**
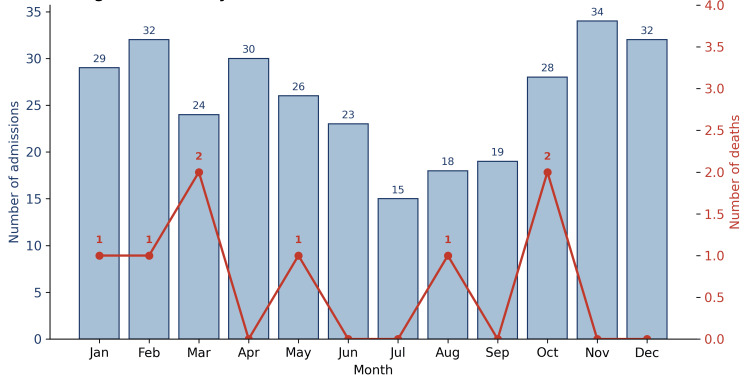
Monthly distribution of PICU admissions (bars) and in-PICU deaths (line) during 2024.

Figure 1 shows PICU admissions by disease category. Figure 2 shows the top 15 admission diagnoses. Figure 3 shows the distribution of PICU admission sources. Figure 4 shows in-PICU mortality stratified by invasive mechanical ventilation status. Figure 5 shows in-PICU mortality by age group. Figure 6 shows the distribution of in-PICU outcomes. Figure 7 shows in-PICU mortality stratified by admission source. Figure 8 shows the case-fatality rate by disease category. Figure 9 shows the monthly distribution of PICU admissions (bars) and in-PICU deaths (line) during 2024.

## Discussion

This study provides contemporary data on admission patterns, short-term outcomes, and mortality-associated factors in a mixed tertiary PICU in northwest Saudi Arabia. Respiratory and metabolic conditions accounted for most admissions, overall mortality was low, and deaths were concentrated among children requiring invasive support or admitted through higher-risk pathways.

An important interpretive point strengthened in the revised manuscript is that the study PICU did not function solely as a destination for mechanically ventilated children. Instead, the dataset shows substantial use of the PICU for close observation, metabolic stabilization, noninvasive respiratory support, and postoperative monitoring. This broader admission threshold likely contributed to the relatively low crude mortality rate and should be considered when comparing outcomes across institutions.

The predominance of respiratory disease in this cohort is consistent with modern epidemiologic data showing that respiratory conditions remain a major driver of PICU utilization [1]. Asthma exacerbation, bronchiolitis, and pneumonia were prominent in the present study, mirroring findings from other PICU cohorts in which respiratory failure and pulmonary disease are frequent reasons for critical care admission [4,9,10]. The low mortality observed among respiratory cases in the current cohort likely reflects the reversibility of many pediatric respiratory illnesses when timely monitoring and supportive therapy are available.

A notable feature of this cohort was the substantial burden of metabolic and observation admissions, particularly diabetic ketoacidosis and close monitoring. This pattern suggests that the PICU in our center functions not only as a unit for invasive organ support but also as a high-dependency environment for early escalation, close surveillance, and prevention of clinical deterioration [1,3].

The overall mortality rate of 2.6% compares favorably with rates reported from many resource-limited settings and is close to figures seen in high-resource environments [1,2,7]. In a recent systematic review and meta-analysis, pediatric ICU mortality varied widely across settings, with substantially higher pooled mortality in low-resource regions than in high-income settings [2]. Likewise, single-center reports from Iran and Ethiopia have described mortality rates around 12% or higher, with mortality strongly influenced by organ dysfunction, mechanical ventilation, and severity scores [7,8].

Mechanical ventilation was the strongest factor associated with mortality in this study. This is consistent with previous work showing that invasive respiratory support is a strong marker of severity in critically ill children [6-10]. The association observed in our study should not be interpreted as causal; rather, the need for invasive ventilation likely reflects underlying physiologic instability, severe respiratory compromise, or multisystem disease.

The admission source was also significantly associated with mortality. Children admitted via external transport or directly from the operating room had substantially higher mortality than those admitted from the emergency department or inpatient wards. This finding is aligned with both regional and international literature [5,11]. These findings highlight the importance of structured pre-PICU stabilization, early recognition of deterioration onwards, and streamlined transfer pathways.

Although age was not statistically associated with mortality in our cohort, infants younger than one year contributed a disproportionate share of deaths. Similar observations have been reported elsewhere, where younger children often have reduced physiologic reserve and are more vulnerable to severe infection, respiratory failure, and rapid decompensation [2,6,7]. Given the small number of deaths in our study, the lack of statistical significance likely reflects limited power rather than the absence of a clinically meaningful trend.

The distinction between admission diagnosis and immediate indication for PICU admission also improves interpretation of the findings. For example, a child with bronchiolitis, pneumonia, congenital heart disease, or postoperative status may enter the PICU because of respiratory distress, shock, metabolic derangement, neurologic monitoring needs, or a requirement for close observation rather than because of a diagnosis label alone. Presenting both diagnosis and admission indication, therefore, offers a more clinically meaningful description of PICU workload and acuity.

An important observation in the current cohort is that mortality was not concentrated in the most common diagnostic groups. Respiratory and metabolic conditions comprised most admissions, yet deaths occurred across lower-frequency, higher-acuity categories, including cardiac disease and complex medical conditions. This pattern is consistent with studies of PICU nonsurvivors showing that death is often driven by severity, comorbidity, and organ dysfunction rather than by the most frequent admission diagnosis alone [8,9,12].

The median PICU stay in this cohort was short at 3 days, likely reflecting a large number of lower-acuity admissions for close monitoring or reversible disease. Nevertheless, published evidence indicates that prolonged PICU stay is associated with increased mortality, greater nosocomial burden, and higher resource consumption [13,14].

Our findings also have implications for benchmarking and quality improvement in Saudi PICUs. National and regional literature has documented heterogeneous case mix and outcomes across Saudi centers, including sepsis cohorts, pulmonary hemorrhage cohorts, and oncology-specific PICU populations [15-17].

The absence of standardized severity-of-illness scores such as PRISM or PIM is an important limitation, but it also identifies a concrete opportunity for future improvement. PRISM III and related models have shown useful prognostic performance in Saudi and regional PICUs, and several studies have linked higher PRISM or organ dysfunction scores with mortality [7,18,19].

Beyond short-term survival, the literature increasingly emphasizes the long-term consequences of pediatric critical illness. Children surviving PICU admission may experience physical, cognitive, emotional, and social sequelae described within the post-intensive care syndrome in pediatrics framework [20]. A rapidly expanding body of literature now addresses child and family outcomes after PICU admission, including psychological burden among caregivers and functional limitations in survivors [20-23].

This study has several limitations. First, its retrospective single-center design limits causal inference and external generalizability. Second, only eight deaths occurred, limiting statistical power and precluding multivariable modeling. Third, the cohort combined medical, surgical, and neonatal-age patients in a single PICU analysis, which introduces heterogeneity in disease profile, intervention needs, and complication patterns. Fourth, the retrospective dataset did not contain a standardized comorbidity field that allowed reliable classification of underlying chronic illness or newly detected chronic disease across all admissions. Fifth, discharge disability status was taken from routine chart documentation rather than a validated functional outcome tool, so this variable should be interpreted cautiously. Sixth, standardized severity-of-illness scores such as PRISM or PIM were not available, restricting adjustment for baseline severity. Finally, long-term outcomes, functional recovery, and family-centered outcomes were not systematically captured.

Despite these limitations, the study has important strengths. It included all eligible PICU admissions over a full calendar year, captured a contemporary cohort from an underreported Saudi region, and described both diagnostic categories and practical admission indications. The findings therefore provide center-specific benchmark data and identify concrete areas for local quality-improvement initiatives in triage, transfer, respiratory support, and outcome monitoring.

## Conclusions

In this tertiary-care PICU in Tabuk, Saudi Arabia, respiratory and metabolic conditions were the leading causes of admission, and overall mortality was low. However, interpretation of this low mortality should take into account the mixed-acuity admission profile, including children admitted for close monitoring, metabolic stabilization, postoperative care, and noninvasive respiratory support. Deaths were concentrated among children requiring invasive mechanical ventilation and those admitted through transport or perioperative pathways. These findings support efforts to strengthen early risk recognition, optimize transfer and perioperative stabilization, standardize severity scoring, and prospectively capture comorbidity and functional outcomes in future studies.
